# Dynamic change of acetabular component position in total hip arthroplasty based on the spinopelvic classification: a prospective radiographic study

**DOI:** 10.1007/s00590-024-04079-2

**Published:** 2024-08-29

**Authors:** Shigeo Hagiwara, Yuya Kawarai, Junichi Nakamura, Yuki Shiko, Rui Hirasawa, Seiji Ohtori

**Affiliations:** 1 Department of Orthopaedics Surgery, Graduate School of Medicine, Chiba University, 1-8-1 Inohana, Chuo-Ku, Chiba, 260-8670 Japan; 2 Biostatistics Section, Clinical Research Center, Chiba University Hospital, Chiba, Japan

**Keywords:** Spinal alignment, Total hip arthroplasty, Dislocation, Risk factor, Spinal flexibility

## Abstract

**Purpose:**

Recent studies have proposed optimizing the position of the acetabular component based on spinal deformity and stiffness classification to avoid mechanical complication after total hip arthroplasty (THA). The aim of this study was to characterize the dynamic changes in cup alignment post-THA based on the spinopelvic classification and to evaluate the efficacy of cup placement of preventing dislocation.

**Methods:**

This prospective study included a total of 169 consecutive patients awaiting THA who were classified into four groups based on spinal deformity (pelvic incidence minus lumbar lordosis) and spinal stiffness (change in sacral slope from the standing to seated positions). The cups were aligned based on the group with fluoroscopy. Additionally, postoperative radiographic inclination (RI), radiographic anteversion (RA) in standard anteroposterior radiograph, and lateral anteinclination (AI) in sitting and standing positions were measured. The cumulative incidence of dislocation was evaluated at a follow-up two years post-THA.

**Result:**

RA was significantly greater in the group with normal spine alignment and stiff spine than in other groups (*P* = 0.0006), and AI in the sitting position was lower than in other groups (*P* = 0.012). Standing AI did not significantly differ between the groups. One posterior dislocation occurred during the study period (0.6%).

**Conclusion:**

AI in the sitting position was lower in patients with normal spinal alignment and stiff spine despite larger RA in the standard anteroposterior radiographs. Consequently, with more anteversion in the normal spinal alignment and stiff spine group, spinopelvic parameters can help guide cup placement to prevent short-term dislocation post-THA.

## Introduction

Total hip arthroplasty (THA) is the gold standard treatment for advanced hip osteoarthritis. Despite high patient satisfaction, dislocation can occur warranting revision THA [1]. As implant impingement is a major cause of dislocation, component position is considered critical to maintain THA stability [2]. To avoid dislocation, Lewinnek et al. proposed angles of 40° ± 10° and 15° ± 10° for the lateral opening and anteversion, respectively, as a “safe zone” to guide the acetabular component, which remains a standard target among clinicians [3]. However, recent studies considering spinal deformity and stiffness as risk factors for dislocation have reported cases of dislocations within the safe zone [4, 5]. As anterior pelvic tilt reduces cup anteversion and leads to anterior impingement in hip flexion, and posterior pelvic tilt increases the likelihood of posterior impingement in hip extension [6], spinal alignment and stiffness caused by deformity or fusion may increase the risk of dislocation [7].

To avoid impingement, several recent studies have proposed using spinal deformity and stiffness classifications to optimize placement of the acetabular component [7–9]. Although some studies have reported that patient-specific placement based on the spinopelvic parameters of the cup can reduce dislocation [10, 11], postoperative radiographic evaluations of cup alignment and the dynamic risk of impingement are scarce. Therefore, whether such cup placement avoids impingement and the consequent dislocation remains unclear.

The primary purpose of this study was to characterize dynamic changes to cup alignment post-THA based on the spinopelvic classification. The secondary purpose was to evaluate the clinical efficacy of the cup placement based on the spinopelvic classification for preventing impingement leading to dislocation.

## Materials and methods

### Patients

This was a prospective clinical and radiographic research of consecutive subjects awaiting THA in our institute between January 2019 and December 2021. A total of 240 in patients with 258 hips underwent primary THA during this period. Inclusion criteria were adult patients with preoperative lateral spinopelvic imaging in standing and sitting positions. Exclusion criteria were less than two-year follow-up following THA; a previous history of hip surgery including THA; osteotomy and osteosynthesis; severe deformity of the hip for radiographic evolution development (severe dysplasia Crowe III or IV and protrusion acetabuli); cemented acetabula cup; neurological comorbidities affecting pelvic alignment; major contracture or ankylosis affecting pelvic alignment; previous history of spinal compression fracture; previous history of spine surgery; and inability for the radiographic evaluation. The previously operated side was radiologically evaluated for bilateral cases to avoid errors in alignment measurement. The cumulative incidence of dislocation during the follow-up period was evaluated. The study protocol was performed in compliance with the Helsinki Declaration. Appropriate institutional review board approval was obtained. The patients gave written informed consent before any procedures were performed.

### Surgical technique and implants

**Fig. 1:**
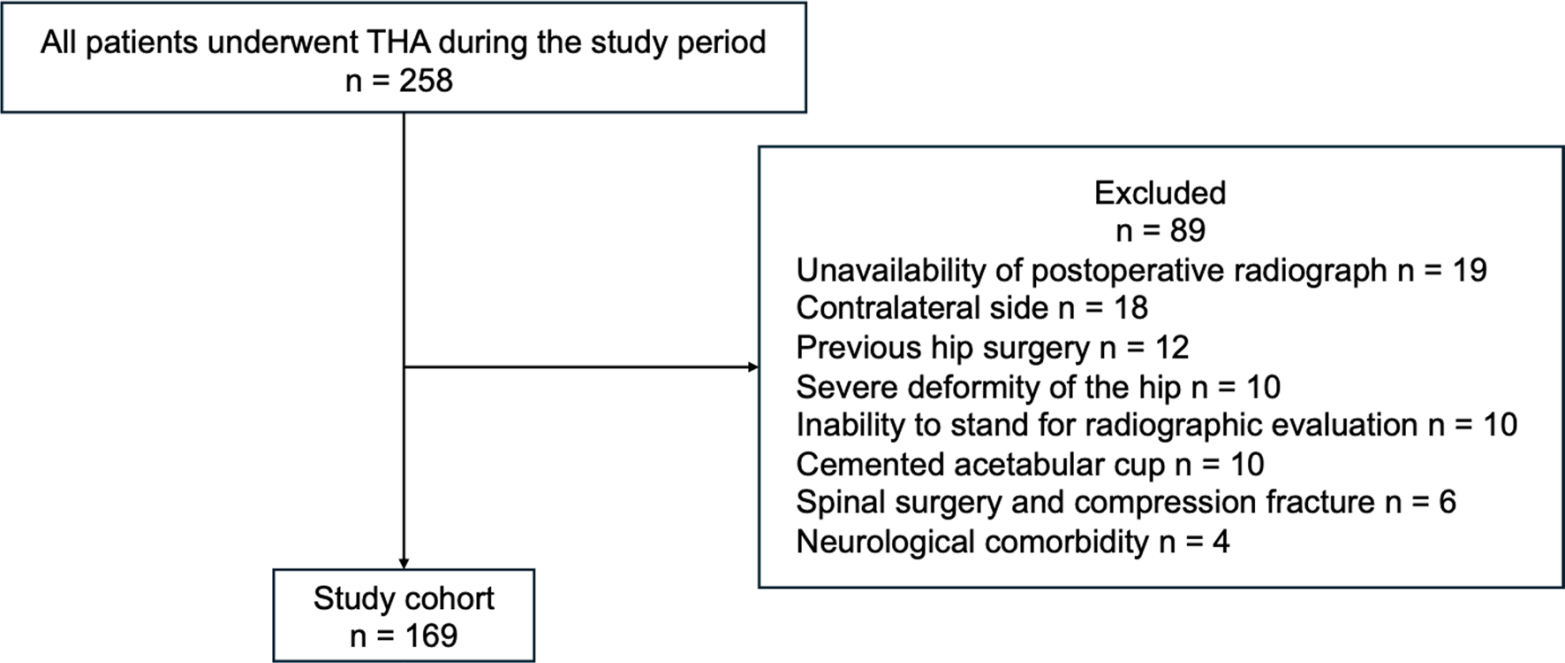
Study flow chart

**Fig. 2:**
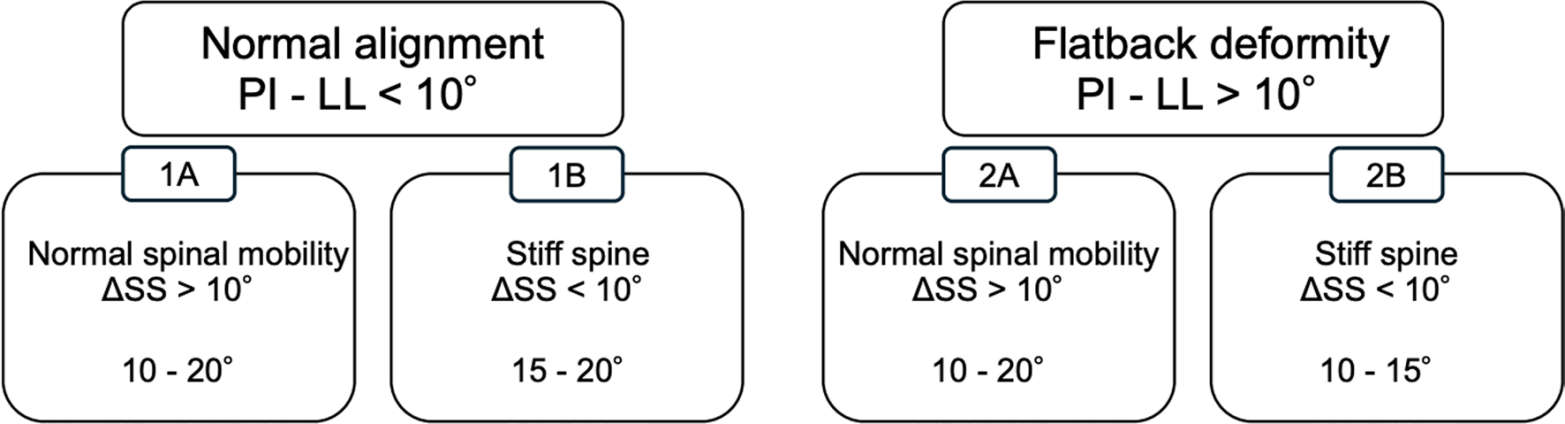
Categories in the hip–spine classification and anteversion target, 1A) normal spinal alignment (pelvic incidence (PI) minus lumbar lordosis (LL) < 10°) and normal spinal mobility (> 10° change in sacral slope (SS) from standing to sitting), 1B) normal spinal alignment (PI minus LL < 10°) and stiff spine (< 10° change in SS from standing to sitting), 2A) flatback deformity (PI minus LL < 10°) and normal spinal mobility (> 10° change in SS from standing to sitting), 2B) flatback deformity (PI minus LL < 10°) and stiff spine (< 10° change in SS from standing to sitting)

A total of 169 hips (women: 136) with an average age of 65 (range, 30–88) were identified (Fig. 1, Table 1). According to the previously proposed algorithm, the patients were classified into four groups (1A–2B) based on preoperative spinopelvic parameters (Fig. 2) [9]. The classification was determined based on the presence of spinal deformity and spinal stiffness. Spinal deformity was defined by a greater than 10° difference between pelvic incidence (PI) and lumbar lordosis (LL). Spinal stiffness was defined by a less than 10° change in sacral slope (SS) from the standing to seated positions. The cup was then aligned according to the group (Fig. 2). In this series, the target radiographic anteversion (RA) was 15° ± 10° within the Lewinnek “safe zone” [3]. Each THA was performed using a direct anterior approach in the supine position by three board-certified senior surgeons (NJ, HS, and KY) with a fluoroscopy guide for settlement of the acetabular cup alignment. The components were selected based on bone quality and morphology. The SQRUM cup (Kyocera, Kyoto) was used in 122 hips, the R3 cup (Smith & Nephew Orthopaedics, Memphis, Tennessee) in 31 hips, and the Trident cup (Stryker, Mahwah, New Jersey) in 16 hips.

**Table 1:** Patient characteristic

	1A (n = 54)	1B (n = 30)	2A (n = 43)	2B (n = 42)	*P*-value
Age (years)	60.4 (11.80)	65.2 (11.75)	66.3 (12.69)	69.2 (11.17)	0.0043
Sex (female), n (%)	42 (77.8)	24 (80.0)	38 (88.4)	32 (76.2)	0.48
Body mass index (kg/m^2^)	24.6 (4.13)	22.4 (3.24)	26.3 (4.43)	24.1 (4.56)	0.0022
*Diagnosis*, *n* (%)					0.13
OA	38 (70.4)	24 (80.0)	39 (90.7)	38 (90.5)	
ONFH	14 (25.9)	5 (16.7)	3 (7.0)	3 (7.1)	
RA	2 (3.7)	1 (3.3)	1 (2.3)	1 (2.4)	
PI minus LL	− 1.5 (8.00)	2.6 (5.24)	19.4 (9.18)	21.5 (9.94)	< 0.0001
ΔSS	21.5 (9.23)	3.6 (4.85)	19.8 (8.26)	3.9 (4.53)	< 0.0001

Values are reported as mean (standard deviation)

OA, osteoarthritis; ONFH, osteonecrosis of femoral head; RA, rheumatoid arthritis; PI minus LL, pelvic incidence minus lumbar lordosis; SS, sacral slope; ΔSS, change in SS from the standing to seated positions

### Radiographic evaluation

All patients underwent radiographic assessment of the whole lateral spine and pelvis in standing and sitting positions as well as standard anteroposterior radiograph of the pelvis within one month prior to and three months post-THA to avoid spinal degeneration and compression fracture. The standing and sitting radiographs were taken following a previously described standardized procedure [12].

**Fig. 3:**
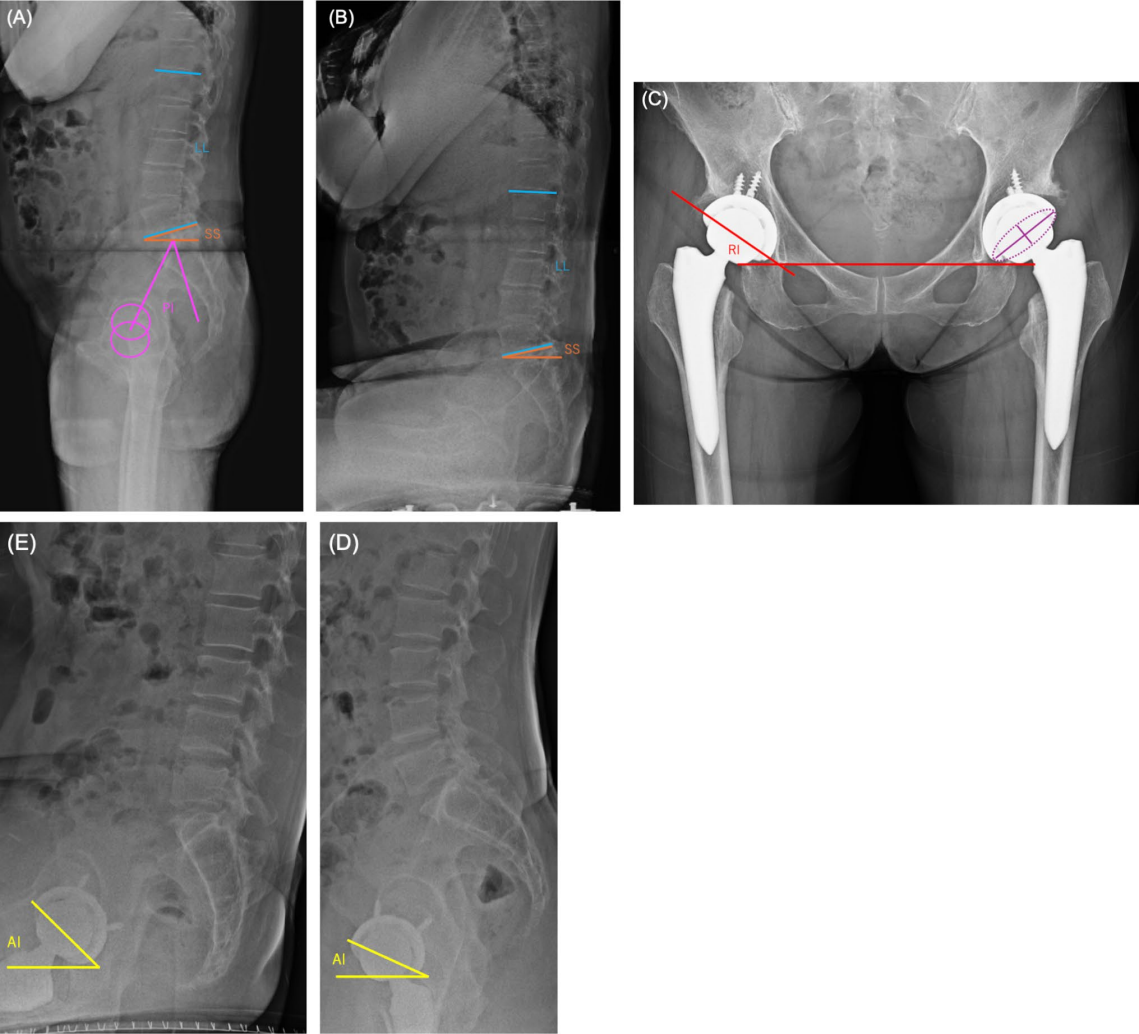
Lateral view showing the spinopelvic parameters. The pelvic incidence (PI) is the angle between the line perpendicular to the sacral endplate and the line connecting the middle of the sacral endplate to the midpoint of the bicoxofemoral axis (pink). The sacral slope (SS) is the angle between the horizontal plane and the sacral endplate (orange). The lumbar lordosis (LL) is the angle between the superior endplate of L1 and sacral endplate (blue). (**A** and **B**), Radiographic inclination (RI) is the angle between a vertical line and a line through the long axis of its ellipse (red) on the anteroposterior plane. Radiographic anteversion (RA) is the arcsin of the short axis of the ellipse drawn perpendicular to the long axis of the component or maximum diameter (purple). (**C**) Anteinclination (AI) is the angle between a vertical line and a line through the long axis of its ellipse (yellow) on the sagittal plane. (**D** and **E**)

The radiographic measurements were performed by two senior joint reconstruction surgeons (YK and HS) independently and were then averaged. Preoperative PI, sacral slope (SS), and LL were measured [13]. The postoperative radiographic alignment of the acetabular component, radiographic inclination (RI), RA in a standard AP radiograph, and lateral anteinclination (AI) in sitting and standing positions were also measured using HOPE LifeMark-PACS (Fujitsu, Kawasaki, Japan) (Fig. 3) [14]. AI in the sitting position was used as the surrogate variable for anterior impingement, and AI in the standing position was used for the posterior impingement. Internal consistency and reliability were assessed using intraclass correlation coefficients (ICCs). The cumulative incidence of dislocation during the follow-up period was evaluated.

### Statistical assessment

Descriptive data were described by means, range, and standard deviation. Demographic data and radiographic parameters were compared between categories using one-way analysis of variance followed by a Tukey–Kramer correction. Categorical data (sex and femoral head diameter) were analysed with a chi-square test. To compare preoperative and postoperative SS, a paired *t*-test was used. The ICC for the interobserver reliability of the radiological parameters was calculated. An ICC value of 1 was considered as perfect reliability, whereas > 0.80, > 0.60, and > 0.40 were considered as very good, good, and moderate reliability, respectively [15]. Statistical analyses were performed with JMP (SAS Institute Inc., Cary, NC, USA). A *P*-value of < 0.05 was considered significant.

## Results

**Fig. 4:**
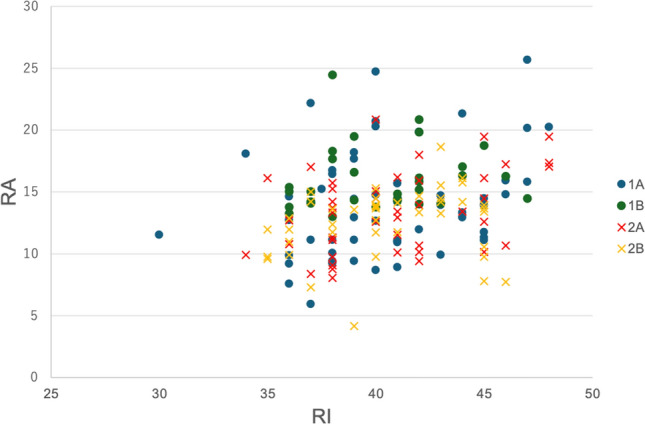
Scatter plot of the distribution of the cup placement angles. 1A, blue circle; 1B, green circle; 2A, red cross; 2B yellow cross RI, radiographic inclination; RA, radiographic anteversion

After radiographic classification of 169 cases, there were 54 patients (31.9%) in group 1A, 30 (17.8%) in group 1B, 43 (25.4%) in group 2A, and 42 (24.9%) in group 2B. Patient demographic and radiographic data are shown in Tables 1 and 2, respectively. There was a significant difference in age and body mass index between the groups (*P* = 0.0043 and 0.0022, respectively). The acetabular components were positioned within the safe zone in 98.8% (167/169) of hips (Fig. 4). RA in group 1B was significantly higher than in groups 2A and 2B (*P* = 0.012 and 0.0003, respectively). The sitting AI in group 1B was significantly lower than in groups 1A and 2A (*P* = 0.0064 and 0.0024, respectively), whereas standing AI was not significantly different between the groups.

**Table 2:** Radiographic data

	1A (n = 54)	1B (n = 30)	2A (n = 43)	2B (n = 42)	*P*-value
RI	40.5 (3.80)	40.3 (3.14)	40.8 (3.65)	40.5 (3.34)	0.93
RA	14.1 (4.43)	16.0 (2.59)	13.5 (3.29)	12.6 (2.75)	0.0006
Sitting AI	48.0 (11.18)	41.2 (8.51)	49.1 (10.81)	45.3 (11.64)	0.012
Standing AI	28.2 (6.35)	30.0 (6.92)	28.1 (6.41)	30.9 (7.47)	0.15
*Femoral head size*					0.69
28 mm	2 (3.7)	0 (0.0)	1 (2.3)	1 (2.4)	
32 mm	21 (38.9)	10 (33.3)	13 (30.2)	10 (23.8)	
36 mm	31 (57.4)	20 (66.7)	29 (67.4)	31 (73.8)	
*Sitting SS*					
Preoperative	11.0 (11.88)	23.5 (8.72)	5.8 (10.04)	15.1 (10.18)	< 0.0001
Postoperative	12.8 (11.46)	19.4 (9.52)	9.1 (9.52)	12.6 (10.57)	0.0009
*Standing SS*					
Preoperative	32.5 (9.23)	27.0 (6.69)	25.6 (10.83)	19.0 (10.14)	< 0.0001
Postoperative	29.8 (8.62)	28.2 (6.33)	22.7 (11.66)	20.2 (10.50)	< 0.0001

Values are reported as mean (standard deviation)

RI, radiographic inclination; RA, radiographic anteversion; AI, anteinclination; SS, sacral slope

Preoperatively, SS in the sitting position in group 1B was significantly higher than in groups 1A, 2A, and 2B (*P* < 0.0001, *P* < 0.0001, and *P* = 0.0011, respectively), whereas postoperative SS in the sitting position in group 1B was significantly higher than in groups 1A, 2A, and 2B (*P* = 0.0063, *P* < 0.0001, and *P* = 0.0069, respectively). Conversely, preoperatively, SS in the standing position in group 2A was lower than in group 1A (*P* = 0.0005) and was lower in group 2B than in groups 1A and 1B (*P* < 0.0001 and *P* = 0.0006, respectively), whereas postoperative SS in the standing position in group 2A was lower than in groups 1A and 1B (*P* = 0.0004 and 0.017) and lower in group 2B than in groups 1A and 1B (*P* < 0.0001 and *P* = 0.0006, respectively).

The interobserver ICC of the radiological measurement was very good (0.98 for RI, 0.93 for RA, 0.92 for PI, 0.99 for LL, 0.92 for sitting SS, 0.91 for standing SS, 0.99 for sitting AI, and 0.93 for standing AI). Except for the three dropout cases, one posterior dislocation occurred during the follow-up period (0.6%). The case was included in this study and classified into group 1B. Overall, in all the groups, dislocation rate was low.

## Discussion

This study investigated dynamic changes in acetabula component alignment post-THA based on the spinopelvic classification and its clinical effectiveness in preventing dislocation. The radiographic evaluation was reliable, and the dislocation rate was low (0.6%). In group 1B, despite the cup anteversion being the largest, the sitting AI, the surrogate variable for anterior impingement, was the lowest.

Dislocation after THA remains one of the most common and expensive complications [16]. Lewinnek et al. [3] evaluated dislocation in a series of 300 THA and proposed the “safe zone” as a relatively safe orientation range for the cup, which has long been the standard range for cup placement. However, a recent retrospective study reported that in most dislocated THAs the cup was aligned within the safe zone [4]. Other recent studies have stated that spinal imbalance, stiffness, hypermobility, and spinal fusion are risk factors for dislocation post-THA [17]. Previous studies have reported a higher likelihood of posterior hip impingement with extension in patients with a flat back and stiff spine [6], the occurrence of anterior hip impingement in patients with normal alignment and stiff spine in hip flexion [6], and that an anteriorly tilted pelvis in the sitting position reduces cup anteversion and can lead to dislocation [7]. Therefore, it has been proposed that spinal deformity and stiffness classification should guide placement of the acetabular component [8, 9]. Moreover, lateral AI has been recently used as an indicator of stability post-THA [6].

Here, target anteversion ranged from 5°–25° to ensure the cup was within the safe zone in all groups. Using a direct anterior approach with fluoroscopy, the cup was aligned mostly as planned in each group. In addition to the high accuracy of the cup placement, this approach may reduce dislocation rate [18]. Although robotic-assisted or navigation systems may more accurately place the acetabular component, the spinal parameter-based classification system with fluoroscopy-guided THA could enhance stability.

The cup anteversion in the 1B group was the largest among the groups, whereas the sitting AI was the lowest. A radiographic study found a correlation between SS and functional pelvic tilt [19]. Loppini et al. suggested adjusting the cup anteversion based on SS. In this study, the sitting SS in group 1B was the highest among the groups both pre- and postoperatively. An anteriorly tilted pelvis with a stiff spine can contribute to the smaller sitting AI. Although there was no significant difference, dislocation occurred in group 1B. Hence, a larger anteversion may be needed for more stability.

Another radiographic study revealed that the prevalence of the flatback spinal deformity was associated with increasing age and posterior pelvic tilt, which can lead to the risk of anterior hip dislocation [20]. In patients with flatback and stiff spine, the cup anteversion should be low to avoid anterior dislocation [9]. The anteversion in this study was lowest in group 2B, and standing AI was not significantly different between the groups. Moreover, cases of dislocation were not observed in the patients with the flatback spine; the target anteversion may thus be reasonable.

There are several limitations to this study. First, we used the standing and sitting AI as a surrogate variable for impingement, not actual dislocation. Because several factors contribute to hip dislocation, it is unclear whether our data is related with a real risk of dislocation. However, the surrogate was reasonable because posterior dislocation occurred in the group with the lowest sitting AI. This study evaluated dislocation within two years post-THA; therefore, our data comprises only short-term risk of impingement. Second, demographic data were not matched between the groups as spinal alignment and stiffness are associated with age. Potential bias regarding dislocation could not be eliminated. Finally, we used lateral spinopelvic X-ray instead of the EOS system, which can affect radiographic measurements. However, the interobserver consistency and reliabilities of our radiological analysis were very good.

In conclusion, our study suggests that patients with normal spinal alignment and stiff spine had lower cup anteversion in the sitting position despite larger RA in a standard AP radiograph. Thus, with more anteversion in the normal spinal alignment and stiff spine group, spinopelvic parameters can be used to guide cup placement to prevent short-term dislocation after THA.
